# Perceptions of Cognitive Training Games and Assessment Technologies for Dementia: Acceptability Study With Patient and Public Involvement Workshops

**DOI:** 10.2196/32489

**Published:** 2022-06-20

**Authors:** Kyle Harrington, Michael P Craven, Max L Wilson, Aleksandra Landowska

**Affiliations:** 1 NIHR MindTech MedTech Co-operative Institute of Mental Health Nottingham United Kingdom; 2 School of Medicine Faculty of Medicine and Health Sciences University of Nottingham Nottingham United Kingdom; 3 NIHR Nottingham Biomedical Research Centre University of Nottingham Nottingham United Kingdom; 4 Human Factors Research Group Faculty of Engineering University of Nottingham Nottingham United Kingdom; 5 Mixed Reality Lab School of Computer Science University of Nottingham Nottingham United Kingdom

**Keywords:** dementia, cognitive assessment, cognitive training, serious games, patient and public involvement

## Abstract

**Background:**

Cognitive training and assessment technologies offer the promise of dementia risk reduction and a more timely diagnosis of dementia, respectively. Cognitive training games may help reduce the lifetime risk of dementia by helping to build cognitive reserve, whereas cognitive assessment technologies offer the opportunity for a more convenient approach to early detection or screening.

**Objective:**

This study aims to elicit perspectives of potential end users on factors related to the acceptability of cognitive training games and assessment technologies, including their opinions on the meaningfulness of measurement of cognition, barriers to and facilitators of adoption, motivations to use games, and interrelationships with existing health care infrastructure.

**Methods:**

Four linked workshops were conducted with the same group, each focusing on a specific topic: meaningful improvement, learning and motivation, trust in digital diagnosis, and barriers to technology adoption. Participants in the workshops included local involvement team members acting as facilitators and those recruited via Join Dementia Research through a purposive selection and volunteer sampling method. Group activities were recorded, and transcripts were analyzed using thematic analysis with a combination of a priori and data-driven themes. Using a mixed methods approach, we investigated the relationships between the categories of the Capability, Opportunity, and Motivation–Behavior change model along with data-driven themes by measuring the *φ* coefficient between coded excerpts and ensuring the reliability of our coding scheme by using independent reviewers and assessing interrater reliability. Finally, we explored these themes and their relationships to address our research objectives.

**Results:**

In addition to discussions around the capability, motivation, and opportunity categories, several important themes emerged during the workshops: family and friends, cognition and mood, work and hobbies, and technology. Group participants mentioned the importance of functional and objective measures of cognitive change, the social aspect of activities as a motivating factor, and the opportunities and potential shortcomings of digital health care provision. Our quantitative results indicated at least moderate agreement on all but one of the coding schemes and good independence of our coding categories. Positive and statistically significant *φ* coefficients were observed between several coding themes between categories, including a relatively strong positive *φ* coefficient between capability and cognition (0.468; *P*<.001).

**Conclusions:**

The implications for researchers and technology developers include assessing how cognitive training and screening pathways would integrate into existing health care systems; however, further work needs to be undertaken to address barriers to adoption and the potential real-world impact of cognitive training and screening technologies.

**International Registered Report Identifier (IRRID):**

RR2-10.1007/978-3-030-49065-2_4

## Introduction

### Background

The World Health Organization has estimated the number of people living with dementia worldwide to be approximately 55 million and the total societal cost of dementia to be approximately US $2.8 trillion [1]. These figures are expected to rise with the increase in life expectancy; however, over one-third of these cases are thought to be preventable by reducing modifiable risk factors [2]. As such, in recent years, there has been growing interest in the application of digital technologies designed to promote healthy lifestyles, particularly in the prevention, management, and mitigation of dementia [3-6]. There is a growing evidence base indicating that healthy lifestyle choices such as exercising, maintaining social engagement, reducing smoking, and engaging in cognitively stimulating activities may help to reduce the overall lifetime risk of dementia [7,8]. Lifestyle factors such as occupation, leisure activities, and educational attainment may help to build *cognitive reserve*, which could help to mitigate or modify the clinical expression of dementia despite underlying neuropathology [9]. Other evidence suggests that certain cognitive domains may be amenable to training [10,11]. Despite this, the extent to which these technologies may help improve cognition or reduce the overall lifetime risk of dementia is debated, and the evidence remains inconclusive [12-15].

Concurrently, cognitive screening offers the potential for early intervention and support but can often lead to frustration or stress and affect patient dignity [16]. There are still challenges around the acceptability and uptake of digital technologies, which are designed to improve or assess cognition [17], including concerns about privacy, decision-making, dignity, and liberty in the use of technology for people living with dementia [18].

The inclusion of gamified cognitive training interventions could enhance motivation, and positive mood and improve assessment [19]. Increasing engagement and adherence to serious games for health often relies on the development and implementation of motivating elements [20], whereas some argue that the use of extrinsic incentives (eg, rewards and penalties) may hinder intrinsic motivation in the long term, potentially affecting self-confidence [21,22].

Lifestyle advice aimed at reducing the modifiable risk factors of dementia and cognitive training and screening regimes falls under the broad category of public health interventions [23], which depend on behavior change to be effective. A widely used model used to understand behavior change interventions is the Capability, Opportunity, and Motivation–Behavior (COM-B) model developed by Michie et al [24]. The model identifies capability, motivation, and opportunity as the primary drivers of behavior. Using this framework, public health behavior change interventions can be systematically evaluated, and appropriate strategies can be developed to maximize the effectiveness of interventions.

Interventions designed to form part of a person’s daily routine need to ensure that there is involvement from patients and the public during the testing and evaluation of cognitive technologies to ensure that the technology is being used as intended and increase uptake [25].

Patient and public involvement (PPI) is advocated in health care research for many reasons; it may help generate research opportunities, identify research priorities, lead to better research methodology, and improve the communication and application of findings [26]. PPI research is conducted *with* or *by* patients and the public rather than being conducted *on* them, *about* them, or *for* them [27,28]. There has been growing acknowledgment of the importance of involving patients and the public in research that places patients and their experiences at the center. PPI research is of particular importance to dementia research in which it is understood to contribute to the quality, relevance, and ethical conduct of research [29].

### Aims and Objectives

This paper reports on PPI activity that formed a part of an industry-academic partnership, Alzheimer’s Disease: Detect Prevent, led by Brain+, a Danish commercial app developer. Brain+ is developing a suite of app-based technologies designed to help reduce modifiable risk factors of dementia by cognitively stimulating gaming and lifestyle coaching. In addition, it is introducing an element of detection of cognitive deficits through a working memory test. A series of PPI workshops were held to discuss the potential benefits of cognitive training games and assessment technologies related to cognition and capture contextual opinions about the dementia journey, such as support after diagnosis, the role of family and other social relationships, and the practical and ethical factors that arise in the adoption of new health technologies.

We aimed to answer the following research questions:

1. How can cognition be measured in a way that is acceptable and meaningful to people with the experience of dementia?
2. What are the barriers to and facilitators of the adoption of digital cognitive training games and assessment technologies?
3. What factors may affect motivation to use serious games for cognitive assessment and training?
4. What are the potential benefits, drawbacks, and risks of assessment technologies for cognitive impairment, and how may such tools fit within the existing health care infrastructure?

## Methods

### Workshop Procedure

Workshops were conducted physically within conference rooms at the University of Nottingham. The workshops lasted between 10:30 AM and 2 PM for each of the days they were run. Lunch and regular breaks were provided to our participants, and each of our workshops was divided into 2 sessions.

In total, four workshops were conducted between October 2019 and January 2020, each with a focus on a different topic relevant to cognitive training and screening technologies:

1. What counts as meaningful improvement?
2. Learning and motivation
3. Trust in digital diagnosis
4. Barriers to digital inclusion

These topics were developed by the researchers and agreed upon by the research and commercial partners involved in the wider project consortium. The topic of *meaningful improvement* facilitated the exploration of appropriate and patient-centered outcome measures for a concurrent feasibility trial of cognitive training. The topic of *learning and motivation* was chosen for the purposes of investigating motivational strategies and the perceived potential of cognitive training, how at-risk individuals and those with lived experience conceptualize learning *cognitive reserve*, and the importance of cognitively taxing activities to delay the onset of dementia. Our third session on *trust in digital diagnosis* allowed for the elicitation of factors that affect trust in digital diagnosis and screening tools, including the social, environmental, and legislative contexts in which screening and the process of diagnosis occur, as well as attitudes and concerns surrounding the storage, handling, and sharing of personal data. In our concluding session, we sought to identify real-life, contextual, and social factors that may influence technology use with the intention of developing strategies to reach otherwise underrepresented and hard-to-engage populations. A summary of our workshop protocols can be found in Multimedia Appendix 1 and the *Materials* section.

### Materials

All participants, facilitators, and researchers were supplied with name badges to facilitate an informal first-name basis tone for the discussions. Voice recorders were used to record the sessions for subsequent transcription. Two of our workshops included presentations about the topic (1 and 2), and the *trust in digital diagnosis* workshop involved an activity that included the use of UnBias Fairness Toolkit Ideation cards [30].

### Recruitment

Participants were recruited via Join Dementia Research (JDR), a UK-based service that connects people living with dementia and caregivers with researchers for the purposes of conducting dementia research. JDR provides a list of names and contact details based on a set of criteria selected by the researcher. As we were interested in recruiting both people living with dementia and their carers, we decided to target carer-patient dyads. We also wished to discover issues around diagnosis; therefore, dyads without a formal diagnosis of dementia were excluded. Finally, we wanted to investigate issues surrounding the potential of cognitive assessments and cognitive training to facilitate early intervention and recruited participants living with mild dementia for whom cognitive assessment and training may serve a therapeutic or rehabilitative purpose. Using purposive selection, 18 dyads presented on the JDR website results were contacted for recruitment, from which 22% (4/18) agreed to be enrolled. Subsequently, 2 carer-patient dyads took part: one living with dementia participated alone without their partner, and another took part as an individual carer after losing his partner to dementia.

Participants were compensated in line with the INVOLVE guidelines [28], which also included transportation costs. Upon attending each workshop, participants were invited to subsequent workshops.

### Participants

Sessions comprised 6 participants (ie, n=3, 50% carers and n=3, 50% people living with dementia), 2 or 3 group facilitators from the local involvement team, and members of the research team (between 2 and 4 in different sessions). Workshop participants were *experts by experience* because of their lived experience and first-hand knowledge of dementia. Within the participant group in the workshops, there was an equal gender split: the 2 carer-patient dyads were married couples, the individual with dementia was female, and the individual carer was male. Of the people living with dementia, one had working-age Alzheimer, one had vascular dementia, and the third had mixed (Alzheimer with vascular dementia), all describing the severity of their symptoms as mild. They were all aged between 57 and 76 years. Although small, the group was culturally and ethnically diverse, with a range of cultural backgrounds and experiences.

The involvement team facilitators had personal lived experiences and shared awareness of a range of mental and cognitive health conditions and were knowledgeable about digital technologies and the processes of coproduction. As such, they contributed additional knowledge and opinions captured in the focus groups along with those of the enrolled participants. During each session, they ensured that the enrolled participants were comfortable and were there to assist them should they have any difficulty. Facilitators were also involved in reviewing the content of the workshops, proofreading documents and instructions sent ahead of each session, and sense checking the analysis.

### Ethics Approval

Ethical approval was granted by the Faculty of Medicine and Health Sciences Research Ethics Committee (approval number 333-1906). Participants were made aware of the aims and methods of the study and had a chance to ask questions before signing the consent form. All participants met the definition of capacity in line with the legal definition given under the Mental Capacity Act of 2005.

### Analysis

#### Qualitative Analysis

Each of the audio-recorded sessions was transcribed via a third-party contractor before being split into meaningful excerpts, which was achieved by attempting to split the entire transcript into the smallest possible excerpts, which were intelligible without any accompanying text. As such, no two excerpts referred to the same part of the transcript. This created a total of 605 such excerpts.

A 2-stage approach to thematic analysis was used [31,32]; excerpts were initially coded with a priori (theory-driven) themes based on the COM-B behavior change model proposed by Michie et al [24]. This model targets 3 areas of importance in behavior change: capability (the individual’s psychological and physical capacity to engage in the activity), motivation (processes that energize and direct behavior; both planned and habitual), and opportunity (factors that lie outside of the individual, which make behavior possible, prompt it, or present a barrier to it). Once COM-B coding was completed, an additional data-driven coding scheme was devised based on the content of the sessions and issues pertinent to the research project.

Finally, the qualitative validity of our results was assessed by asking the PPI facilitators to comment on our findings before publication, with the aim of increasing the dependability of our results.

#### Quantitative Analysis

Both coding schemes were quantitatively evaluated by another reviewer (coauthor), who coded the excerpts according to the coding scheme during our initial analysis using a small subsection of our excerpts (64/605, 10.6%), which were assessed for consistency using proportional agreement and Cohen κ. This helped ensure the validity and reliability of our findings.

We also looked at the intersections of excerpts coded in our themes, both to ensure that coding schemes within respective coding categories were sufficiently independent, as well as to analyze the relationships between themes of separate coding categories. Best practice dictates that where possible, coding categories should be exclusive [31], indicating that there should be little overlap between separate coding schemes. However, because the analysis used 2 separate coding schemes (one theory driven and another data driven), themes from different schemes do not need to meet this criterion. The *φ* coefficient calculations were conducted to determine the correlation between each theme. This served 2 distinct purposes. First, coefficients for themes *within* respective coding schemes indicate the extent to which each of the thematic categories could be considered independent and exclusive categories. Strong positive correlations would potentially indicate that the themes were not sufficiently independent.

Second, we also wanted to determine the relationship between the various thematic categories *between* the 2 separate frameworks to understand how our data-driven categories were related to broader theoretical determinants of behavior expressed in the COM-B model. Positive correlations indicated the extent to which excerpts matched 2 separate themes simultaneously, whereas negative correlations indicated a decreased likelihood of a single excerpt being coded for both of those categories.

## Results

### Overview

A description of the main research findings can be found in the following sections; more information about the themes and how the excerpts were coded can be found in Multimedia Appendices 2 and 3, along with the coding criteria used by both analysts and example excerpts. Quotations are given using *P* to denote a person with dementia, *C* to denote a carer or spouse, and *F* to denote a facilitator having lived experience of mental or cognitive health problems.

### COM-B Themes

#### Overview

Analysis of the transcripts using the COM-B model revealed several interesting findings. Capability was often viewed with respect to self-care and the tasks of everyday living. Group attendees generally agreed that capability diminishes over time and that a good intervention was one that would lessen the rate of decline and allow people to live independent lives, where they could pursue their hobbies and passions. The theme of opportunity typically revealed several complex and interrelated issues. The theme of opportunity included the health care system and diagnostic pathway, the activities and support available for people living with dementia, cultural and social norms, and how technology may interact with all of the above. Finally, motivation was tied into several areas, including the motivation to play cognitively challenging games and develop new skills later in life and, finally, the importance of appropriate and timely feedback that did not overburden users of cognitive games.

#### Capability

People living with dementia were very much aware of their diminished capabilities because of their cognitive impairment.

##### Impact on Everyday Life

Cognitive difficulties can affect every aspect of a person’s life, including socializing, work commitments, hobbies, social activities, and activities of daily living. A participant said, “I found out that I had Alzheimer’s because I couldn’t do my job any more as a lawyer” and that their “whole life has changed” (P3).

##### Fluctuating Capability

Attendees also mentioned how capability was not static but could be affected by external factors and could fluctuate, thereby affecting people’s abilities to engage in meaningful tasks related to hobbies and the activities of daily living:

It might just be something on that day, there’s an external factor that’s going to have a great deal of influence on that day.

F1

##### Maintaining Active Lifestyles

However, attendees agreed upon the importance of maintaining an active lifestyle to support quality of life and independence. Participants were eager to retain and even build upon their existing capacities, although they typically viewed their ability to learn and retain information as gradually decreasing over time. It was important for our participants to have meaningful things to look forwards to in their lives:

...you need to try and carry on as normal because if you start taking things away from people, they start to feel bad.

P1

##### Risk and Independence

Several excerpts also discussed capability with regard to risk and independence; for example, certain activities were thought to enable people to live independently but could be dangerous when performed by someone with cognitive difficulties. Safety in the home was a concern expressed by one carer in the group, whereas there was an understanding that the capability to remain safe changes over time, and it was indicated that over the course of the progression of dementia, safety may become prioritized over independence:

Appliances are very dangerous, even falling down the stairs can be dangerous.

C3

#### Opportunity

In addition to the definition of opportunity in its positive sense (described previously), we also considered the barriers that lie outside the individual, such as the absence of opportunities, restrictions, coercion, and social pressure.

##### Services and Support

The discussion around opportunity included the availability of local social activities designed for people living with dementia, such as *Singing for the Brain,* and personal activities with friends and family, and participants mentioned several areas where technology may be able to facilitate these tasks, such as navigation tools and personal reminders. They also recounted contact with health services where opportunity was expressed negatively as a lack of support following diagnosis:

I think that when people are diagnosed with cognitive impairment it is quite a shock right, you do not expect it will happen to you even though you know it happens. Then there is no support to help you...

P1

##### The Impact of Cultural Norms

The same participant stressed that an activity they had managed to find and attended with their partner had an assumed shared history that was not relevant for them, having moved from outside the area:

The memory class we didn’t find helpful at all because they were concentrating on the past in Nottingham.

P1

In addition, people felt that cultural norms had, in the past, limited their opportunities and that this belief could be internalized, limiting their own expectations and self-belief. For instance, a person living with dementia said the following about their childhood:

The girls were not really encouraged as much as the boys were.

P1

Other negative evaluations, even at a young age, were thought to have a negative impact on self-perception:

I think there were often subliminal negative reports at school, this boy will never be very good at whatever there was quite a lot of that in school reports, which might have held sway.

C3

A group facilitator agreed as follows:

If you’re told often enough that you can’t do something, you start to believe it.

F1

##### Opportunities Afforded by Technology

New opportunities for support, monitoring, and health care services facilitated by technology were also mentioned. The use of technologies and internet-based platforms was seen as “an opportunity to be proactive” (F3). However, participants were not entirely uncritical of additional opportunities afforded by monitoring technologies for people with dementia and showed some concern about restrictive measures that may hinder privacy and lead to concern about how their data might be used:

You’re being compulsorily monitored, you know, like being tagged almost. It can be an invasion of human rights, I think.

F1

##### Cognitive Screening

Some also expressed concern about being told that they were at risk from a certain condition with no known cure and questioned the need to tell people about something that would cause them a great deal of anxiety. Although cognitive screening affords new opportunities for early diagnosis, the potential for early cognitive assessment was not necessarily seen as a universal good:

They’re now finding ways to check people to say, “This person is possibly going to have it in twenty years' time.” Well, do you tell them twenty years beforehand?

C3

#### Motivation

Many motivating factors were discussed, both in relation to controlling the symptoms of dementia and regarding ambitions more generally.

##### Acquiring New Skills

Many group attendees acquired new skills later in life, including painting, gardening, cooking, use of technology, and being involved in dementia research:

I think that older people that have learnt a computer later in life, I feel my brain is better from all the learning that I have done with a computer.

F1

Cognitive health and cognitive training were viewed in a similar, positive way as physical health, and exercise and cognitive training were considered analogous to “going out for a jog” (C2).

##### Challenging Oneself

Attendees were particularly interested in being challenged by cognitive games, wanted to know whether skills could be improved, thought that having short-term attainable goals was a good motivational factor, and valued resilience in the face of adversity. On numerous occasions, workshop participants mentioned that they knew their cognitive skills would deteriorate over time but felt no point dwelling on the fact and insofar as possible wanted to *carry on* and live their lives to the fullest extent possible:

One thing I have learnt is that to do the things on my own as much as possible is to carry on to be who I am until the day I can’t do it.

P1

##### Gaming Features

Competitive games were also seen as motivational, playing against either friends and relatives or other people worldwide. Activities that provided a level of challenge were seen as both intrinsically motivating factors and opportunities for learning. The participants seemed to value positive feedback given by mobile phone apps:

I think positive feedback is good and I feel because I am doing this diet app at the moment and every day you get a little quiz and if I get 5 out of 5, I feel really good...

C2

##### Notification Fatigue

However, although feedback and other motivational strategies were seen as valuable, if these were seen to place too much of an expectation on people, it was thought that this may have a demotivating impact in the long term. This could be reflective of the broader trend of extrinsic motivation undermining intrinsic motivation in some instances:

I have got an app for my iPad and every time I open it, it tells me, “Is your memory still good?” and then I want to say, “Get off!” Every time I open it it's there, “How is your memory doing?” It tells me every day, that’s what I was saying. [laughter] I don’t want to get rid of the apps completely, but I want it to stop telling me everything.

P2

### Emergent Themes

#### Overview

Emergent themes were devised by considering the main recurrent subject topics and the particular exploratory purposes of our research. The details of the theme descriptions and example excerpts are available in Multimedia Appendix 3. The themes were technology; friends, family, and support; work and hobbies; health care system; and cognition. Performing the second round of analysis and coding the transcripts independently based on specific domain issues allowed for more issues to be identified, which closely aligned with our aims. For instance, the benefits and drawbacks of technology could be identified more easily. Participants saw the value of technology in helping to diagnose and support people living with dementia but were worried that technology may replace human-centered care and that this may have a negative impact on their overall experience because of a lack of support and a lack of holistic understanding. Family, friends, and support networks were seen as integral to a person’s life, either from a spouse helping to detect changes in cognition and enabling them to live independently or from hobby interests or specific support groups. However, it should be noted that people living with dementia did not see themselves as only receiving support but considered the relationship reciprocal and saw value in being a source of support and providing for their friends and family through activities such as cooking and caring. Through this coding category, we were able to highlight that cognition was viewed in concrete and practical terms. Excerpts relating to the health care system revealed diverse experiences of the diagnostic pathway; many felt their care had been *disjointed* and left them feeling isolated.

#### Technology

A variety of views were expressed about technology, its potential role in improving quality of life, and the possibility of digital assessment methods for cognitive impairments. Most of our participants enjoyed cognitively stimulating activities, valued the objective measures of their performance on cognitive tasks, and appreciated the instant feedback afforded by technology.

##### Cognitive Offloading

*Cognitive offloading* strategies, such as diaries and reminders, were also mentioned, although some worried that this may actually lead to deskilling:

Even though we’ve lost our memories, there is still the ability to put the thing in the telephone and get the answer. Which I must say, we do a lot of!

C3

...the phone become our memory. I mean I used to have x number of telephone numbers in my head, and now I can just about remember mine. So, I think, in a way, you know, we're de-skilling ourselves, certainly de-skilling our memory.

F1

##### Ease of Use

Workshop attendees were interested and enthusiastic about understanding the possible benefits of technology for people living with dementia and were optimistic about technology becoming easier to use and more accessible to a wider variety of people:

I am curious as I want to see how the technology can possibly help somebody who is a real technophobe and my husband as well. I am really looking forward to seeing what might be forthcoming.

C2

##### Gaming as a Waste of Time

As mentioned previously, participants also discussed playing games on mobile devices and often enjoyed games that they considered a challenge or games that involved some mental stimulation. However, in general, computer games that were not seen as providing any additional benefit were viewed negatively by members of the group:

I can remember playing Angry Birds once and after about 10 goes I thought this is such a waste of time.

P1

Overall, views on technology were largely positive, and there was general optimism about how technology could improve the lives of people living with dementia, leading to better health outcomes overall. However, as mentioned previously, other views expressed concerns about privacy and deskilling.

#### Family, Friends, and Support

Family, friends, and close personal relationships were particularly important in managing cognitive impairment. Family and friends were seen as sources of support and enablement and were also those best placed to understand changes in cognition.

##### Detecting Cognitive Change

One of the participants indicated that their spouse was able to detect changes in cognition before they became aware of any cognitive issues:

My husband knew before me...

P3

However, it should be noted that those who are in contact with a person living with dementia every day may be less likely to notice gradual changes in cognition, and those who visit more infrequently would be better placed to notice differences:

It is difficult to tell really, I think if you are living with someone, and you are there all the time unless there is a massive change you don’t notice... you only see somebody say once week or however often it is you might notice something more whereas the person living with them wouldn’t.

C3

##### Support and Being Supported

Friends and family were also discussed in the context of helping facilitate activities and hobbies and providing emotional support. However, it should also be noted that this relationship was often seen as reciprocal, and people living with dementia also discussed providing for their friends and family through cooking and housework:

I cook for my daughter...

P1

#### Work and Hobbies

##### Overview

Hobbies and interests were very important to the participant groups. Several participants mentioned discovering new hobbies later in life, and many mentioned actively seeking out groups and activities such as *Singing for the Brain*:

I see a great difference in this as my wife was able to sing and she loved the singing side, which is very well known with dementia.

C3

##### Hobbies as Beneficial

Hobbies that were seen as providing additional benefits to either oneself or others were highly valued. Session attendees frequently mentioned puzzles such as number and word games on their mobile devices but were critical of activities they saw of little value to themselves or other people:

You’ve got to give yourself an interest. Gardening, I’ve found is ideal if you’ve got an allotment, you’ve got a community...

C1

I have been hanging on to that because I have been learning Spanish for 10 years.

C2

For me if it was actually shown to improve something it would make me do it.

P1

The advantages of asynchronous competitive games were also mentioned:

You can play it at any time so you don’t have to sit and play I suppose like the normal game of scrabble, but I suppose you can just abandon it and come back to it later.

C2

##### Technology in the Workplace

Several (but not all) participants were employed when computers and other digital artifacts were introduced in their workplace. However, some mentioned that they were unable to keep up with the pace of change:

We...we had no choice at work, they brought in computers so anything you were booking in or out had to be done on a computer, so you had to learn that. I was never happy with them, but I could find my way round. The one thing I never liked was mobile phones and I still don't. I call them the curse of the twenty-first century!

C1

#### Health Care System

Attendees were, on the whole, very eager to take advantage of therapeutic activities offered by health care and third-party providers, although there was great variability in the support offered:

I think we were lucky because at our surgery as they teach in the practice, so we had loads of stuff coming through like, "This programme is on, that programme is on and are you interested?"

C2

“Go home.” That’s it. No support, nothing, just gave me the books and, “Go home.”

P1

Digital technologies were seen as potentially transformative to health care systems. However, group members did not see technological developments within health care as being able to solve all issues related to dementia. They worried that the health care service was no longer integrated, which could lead to inconsistent treatment, a lack of support, and a breakdown of trust. In addition, there were mixed opinions about the possibility and accuracy of digital cognitive assessments without the presence of qualified clinicians:

a computer will be able to diagnose what we've got better than a surgeon or a doctor. That's the way it's moving.

C3

I just think it's dangerous to see the test and you in some sort of a vacuum as though that's all that there is.

F1

#### Cognition

##### Overview

Participants had an intuitive understanding of changes in their cognition and saw treatments and therapies as a way of reducing the rate of cognitive decline rather than lessening their symptoms:

It only goes one way. If it goes back up again that is not positive because I can assure you it goes that way.

P1

##### Functional Measures of Cognition

Objective, functional, and performance-based measures of cognition were seen as the most appropriate indicators of cognitive issues, and dyads thought that difficulty in learning new things might be an indicator of cognitive decline. There was a general group consensus that other people (rather than the person with dementia themselves) were better able to detect changes in cognition:

...he suddenly realised he had got the map upside down and that would never have happened, it was so out of character and there must of be lots of other little things, but I just thought there is something going on here that is not quite right.

C2

##### Diagnosis and Self-image

A diagnosis of cognitive impairment, dementia, or the risk of these could lead to a lack of confidence and increased anxiety:

Because if you’re given numbers about yourself, and one day the numbers are twenty points lower, whatever those numbers are, if they’re twenty points lower than they were the day before, that could possibly lead to anxiety.

P2

It robbed me of my confidence, and I do not know how to get it back still.

P1

### Interrater Reliability

Coded excerpts were checked for interrater reliability initially by determining the proportion of observed agreement (OPA) and then finally by calculating Cohen κ: capability (OPA=0.762; Cohen *κ*=0.491), opportunity (OPA=0.683; Cohen *κ*=0.368), motivation (OPA=0.746; Cohen *κ*=0.451), technology (OPA=0.921; Cohen *κ*=0.838), friends, family, and support (OPA=0.841; Cohen *κ*=0.624), work and hobbies (OPA=0.794; Cohen *κ*=0.566), cognition (OPA=0.723; Cohen *κ*=0.408), health care system (OPA=0.905; Cohen *κ*=0.670).

### Relationship Between Themes

Table 1 indicates that in general, there was a good level of independence between themes within the same category, with the highest positive *φ* coefficient between 2 themes within the same framework of 0.091 (technology and health care system). There were positive, statistically significant *φ* coefficients between themes belonging to different coding categories (capability and cognition; opportunity and technology; opportunity and health care system; motivation and technology; and motivation, work, and hobbies), indicating the extent to which the COM-B elements mapped onto our data-driven themes.

**Table 1:** The *φ* coefficient between themes to look for relationships.

Themes	Capability	Opportunity	Motivation	Technology	Friends, family, and support	Work and hobbies	Cognition
Opportunity	−0.231^a,b^	—^c^	—	—	—	—	—
Motivation	−0.229^a,b^	−0.279^a,b^	—	—	—	—	—
Technology	−0.234^b^	0.153^a,b^	.0815^d^	—	—	—	—
Friends, family, and support	−0.017	0.073	0.027	−0.122^a,d^	—	—	—
Work and hobbies	0.019	−0.061	0.147^b^	−0.237^a,b^	0.019^a^	—	—
Cognition	0.468^b^	−0.083^d^	−0.184^b^	−0.110^a,d^	−0.057^a^	−0.220^a,b^	—
Health care system	−0.219^b^	0.282^b^	−0.137^b^	0.091^a^^,^^d^	−0.151^a,b^	−0.333^a,b^	−0.034^a^

^a^Coefficients between same framework.

^b^*P*<.001.

^c^Not applicable.

^d^*P*<.05.

## Discussion

### Qualitative Findings

Several interesting findings came from the workshop sessions, with implications for technology designers and policy makers involved in developing serious games and cognitive assessments for people living with dementia.

Technology designers should be considerate of the fact that cognitive assessments might be stressful for some people who worry about cognitive decline. Presenting the results from cognitive assessments without the support of clinicians may cause undue worry about cognition; therefore, this finding agrees with earlier work on the experiences of cognitive screening [33].

Our workshops indicated that people had widely differing experiences of the diagnostic pathway, with some feeling supported and others feeling isolated. Inequalities in the provision of care and care outcomes in dementia are known; however, their determinants are underresearched [34].

An unexpected diagnosis of dementia or cognitive impairment can lead to uncertainty and raise questions regarding identity and autonomy. Previous research indicates that this experience is not uncommon and is often shared by partners of those who have been diagnosed [35]. Therefore, it is vitally important that cognitive screening technology offers options for further support and provides resources that may help counter the potential feeling of disempowerment. Digital technology may offer a way of countering some of these existing inequalities in dementia care; however, equality itself is not guaranteed by the use of digital technologies [36].

Confirming a dementia diagnosis is often a lengthy process [37]; screening technology developers should consider the role they play in the overall diagnostic pathway and how screening may fit into existing health care systems. Technology designers and policy makers must ensure that the results of cognitive assessments taken on at-home devices signpost or link to appropriate clinical support and provide an environment that guides users through the appropriate next steps.

Participants were in favor of using performance-based and objective measures of cognition but raised concerns about the results of these assessments being taken in isolation. However, older patients who are hospitalized and undergo cognitive screening often report being unaware of the significance of screening tests and feel stressed because of the pressure to perform, sometimes evoking feelings of shame and irritation [16].

People were also generally worried that their results would only be a snapshot of their performance at the moment and not necessarily indicative of their cognition in general, and their results could be affected by a variety of factors, not necessarily indicative of dementia. Conversely, it is known in dementia, particularly Lewy bodies and Alzheimer, that cognition does indeed fluctuate, and this may have an impact on clinical diagnosis [38]. Therefore, it is advisable that any cognitive screening or training technologies attempt to take a more holistic and individualized approach, which takes account of personal circumstances and cognitive fluctuation, ensuring that the screening takes into consideration a broad range of factors and not just a single example of performance at any particular time.

Regarding cognitive training aspects, participants were particularly motivated to engage in activities that they felt would be of some benefit to themselves or others; this included cognitively stimulating games and apps. Despite this, participants expressed skepticism about gaming in general. Therefore, cognitive games should relate to and emphasize the potential real-world applications of cognitive training, which may be applicable to other areas of life and should arguably be presented as distinct from games. Equally, people living with dementia and their carers placed great importance on their ability to perform practical tasks as an indicator of cognition. Therefore, cognitive assessments that more easily relate to the activities of daily living (such as the instrumental activities of daily living [39]) may be seen as more acceptable to people at risk of dementia.

When discussing their own hobbies and motivations, the social aspect of pastimes and the importance of community building were frequently mentioned. Most attendees had taken up new hobbies and skills at an older age, often viewing the acquisition of new skills as a way of keeping their mind and body active but also to form new community groups in retirement. Several participants mentioned gaining enjoyment from the socially competitive element of games and the idea of playing asynchronous games against people they knew. Participants mentioned that turn-based competitive games could fit into their own lifestyles without the pressure of an immediate response. App developers may wish to further emphasize and develop the community-building aspects of cognitive training technologies. However, it should be noted that communication abilities may decline with the progression of dementia [40,41]; hence, the social aspects of gaming may not be as relevant to those with severe clinical symptoms.

### Quantitative Findings

When considering the quantitative aspects of our work, we found that the theory-driven analysis framework (COM-B) resulted in less overall interrater reliability than our data-driven framework. Although the framework was a useful taxonomy for understanding the broad theoretical determinants of behaviors related to brain-training and cognitive screening technologies, it resulted in a lower overall interrater agreement. We considered interrater reliability as measured by an observed proportional agreement of ≥0.6 to be low but acceptable for our purposes, 0.7 to be good interrater reliability, and >0.8 to be very good interrater reliability. Using Cohen κ benchmarks put forward by Landis and Koch [42], we observed that agreement on the technology theme was *almost perfect* and that there was substantial agreement on coding of the health care system and friends and family themes; moderate agreement on capability, motivation, and work and hobbies themes; and only fair agreement on the opportunity theme. The lower interrater reliability for the COM-B categories applied to data versus data-driven themes is not surprising.

With reference to the naming conventions suggested by Rea and Parker [43] for analyzing the strengths of association in cross-tabulated data, we took <0.1 to indicate a negligible association, ≥0.1 and <0.2 to indicate a weak association, ≥0.2 and <0.4 to indicate a moderate association, and ≥0.4 and <0.6 to indicate relatively strong association. Capability was most strongly related to cognition (0.468), and this was highly significant (*P*<.001), indicating a relatively strong relationship between participants’ conceptions of the two, a relationship that is well established in the literature [44,45]. Opportunity was most strongly related to the health care system (0.282) and moderately associated; opportunity also had a weak association with technology (0.153), and these relationships were highly significant (*P*<.001), further indicating that participants viewed technology as being able to provide additional opportunities. Motivation was most strongly related to work and hobbies (0.147; *P*<.001), emphasizing the importance of hobbies and leisure activities as motivating factors in the lives of our participants, although opportunity showed a lower correlation with any single emergent theme than either capability or opportunity. This may be because capability is often seen as more individualistic (*within* the person), and motivation is related more to the habits, preferences, and long-term goals of individuals. The strongest positive relationship between the friends and family subtheme and any of the COM-B themes was with opportunity, although this relationship was negligible (0.073) and insignificant. This may be because friends and family were often discussed in the context of health care or shared activities, providing part of a supportive role rather than being the subject of the conversation. Overall, we observed a good level of independence between themes within the 2 coding categories; however, we observed a statistically significant relationship between the health care system and technology themes, although the strength of this association was negligible (0.091).

### Strengths

Using the method described for the PPI workshops, we found that participants were able to talk about the issues presented to them in great detail, with reference to their own personal lives and those close to them. Patient and carer dyads provided added value in understanding by allowing a shared perspective of similar incidents [46], which was a view shared by the researchers, facilitators, and participants involved in this study. In practice, this often meant that carers would add context or fill in details. Workshop attendees were supportive of each other and were willing to openly share their experiences and attitudes about living with dementia, expressing a wide range of views on issues surrounding diagnosis and the potentially transformative impact of technology. Before the workshops, facilitators helped create and moderate the workshop agenda and assisted in reviewing the wording of communications with workshop participants. Facilitators also ensured that all participants were comfortable, both physically and emotionally, throughout the sessions and that everyone was able to contribute.

Using a 2-stage coding system, we were able to map our data-driven themes onto what we considered the key determinants of behavior; however, we acknowledge that even our *data-driven* themes were also, in part, influenced by our research considerations.

Finally, facilitators sense checked our work to improve the dependability of our results [47]. Interrater reliability checks served to establish the credibility and confirmability of our findings [47].

### Limitations

Although the participants reflected a range of different cultural backgrounds, the findings from relatively small workshops may not be generalizable to the population as a whole; caution must be taken in regarding these findings as universally applicable. Although the number of participants was relatively small, we believe that a smaller group setting was beneficial in allowing each participant to express themselves without fear of interruption. The data set that we gained from these workshops also reflected a variety of attitudes, opinions, and circumstances. We do not claim to have reached data saturation on these issues, and there may be other themes related to each of our workshop topics that are yet to be identified. However, we believe that the depth of our discussions (over 10 hours 24 minutes of interview materials), the variety within our workshops (4 separate topics, each split into 2 sessions), and the interpretive status of evidence (confirmed by our checks on interrater reliability) demonstrate that the materials have met data adequacy [48] and that our findings are a reflection of genuine attitudes held among key user and patient groups relating to cognitive training and screening technologies.

Owing to practical considerations of room size and considerations of allowing all participants to contribute to the discussion, we were limited in the number of additional participants that we could recruit. Although we were prepared to have different participants on the 4 days, we encouraged participants who had been involved in earlier workshops to return to subsequent ones; as such, the same group attended all of them. Working with a different group in each of the 4 workshops might have increased the breadth of the viewpoints. However, we believe that this was ultimately a strength of our approach, as participants developed a rapport with each other during the sessions and were consequently willing to share personal experiences about sensitive topics in our later sessions.

The used recruitment method may have led to a selection bias that favored those already interested in dementia research and, hence, people who may be more likely to view these interventions favorably. Involving carers in the sessions raised the possibility of them speaking on behalf of those living with dementia. It could be argued that recruiting dyads limited the representativeness and transferability of our sample. However, in practice, we found that carers would often provide additional contextual information, offer a different perspective on a similar incident, or else provide an example that could be expounded upon. In general, carers were supportive and helped explain things that their spouses may have been struggling with, as well as helping with practical issues such as transportation to the venue.

### Areas for Future Work

Research on serious games for people living with or at risk of cognitive impairment needs to further explore the consequences of technology in relation to the quality of life, digital rights, and overall well-being to facilitate better usability and acceptability [49]. Although we showed participants a brain-training application to contextualize the discussion, the workshop attendees had not themselves been recently engaged in a cognitive training regime and, therefore, could only talk in general terms about the concept and how it would relate to their own experiences. Therefore, future work should aim to elicit the responses of people at risk of dementia who have experienced digitized cognitive training and assessments to garner more specific issues that are likely to emerge because of these technologies.

### Conclusions

Motivation and user attitudes toward cognitive gaming is an underresearched area, and our workshop study, with analysis using the COM-B model and data-driven themes, has revealed a variety of opinions about both cognitive training and digital assessment technologies. More broadly, participants were able to express their opinions well on the opportunities and potential shortcomings of the digital health care provision. Potential facilitators included close support networks as a way of increasing motivation in meaningful activities in the context of cognitive training and, for cognitive assessments, the provision of instant feedback. The growing acceptability and use of technology facilitate both of these activities. End user perceptions of potential benefits included the ability of timely and accurate diagnosis; however, a potential shortcoming was that digital assessments may not take into account the context or the fluctuation of cognition.

This study identified some negative opinions regarding playing games for cognitive health. A potential solution is the enhancement of real-world, social, or community aspects of cognitive gaming. Regarding assessment technologies, concerns were raised about the lack of integration and consistency within the health care system and the lack of support following diagnosis. There were mixed opinions about the utility of cognitive assessment technologies for identifying dementia risk. There were also concerns about privacy and potential misuse of personal data.

Further user-centered research, including PPI activities, will help optimize the design of technologies that promise to improve cognitive health and well-being.

## Appendix

Multimedia Appendix 1 Workshop activities and materials.

Multimedia Appendix 2 The Capability, Opportunity, and Motivation–Behavior model themes.

Multimedia Appendix 3 Emergent themes.

## Abbreviations

COM-B: Capability, Opportunity, and Motivation–Behavior
JDR: Join Dementia Research
NIHR: National Institute for Health and Care Research
POA: proportion of observed agreement
PPI: patient and public involvement
