# Supplementary material for: Perceptions of Cognitive Training Games and Assessment Technologies for Dementia: Acceptability Study With Patient and Public Involvement Workshops
Source: JMIR Serious Games. 2022 Jun 20;10(2):e32489. doi: 10.2196/32489 (PMC9253969; doi:10.2196/32489)
Supplement: Multimedia Appendix 1 [file games_v10i2e32489_app1.docx]

Appendix I Workshop Activities and Materials

# Session 1 - 2/10/2019

## Timetable

| Time | Activity | People |
| --- | --- | --- |
| 9:45 | Facilitators arrive at office | Facilitators |
| 9:45 | Team goes to workshop room | Staff |
| 9:50 | Group ensures room is ready. Information sheets, Expense forms, Consent Forms, Presentation | Staff |
| 10:00 | One researcher waits in car park with parking vouchers. Facilitators wait in SCC reception; other researcher waits in room. | Staff |
| 10:10 | Tea and Coffee arrive. |  |
| 10:30 | Session starts, participants asked to read information sheets and sign consent forms. | All |
| 10:40 | Welcome and ground rules | Facilitators |
| 10:45 | Icebreaker | Researcher 2 |
| 10:50 | Introduction to topic | Researcher 1 |
| 11:00 | Group discussion | Group |
| 12:00 | Lunch |  |
| 12:30 | Introduction to second discussion | Researcher 1 |
| 12:40 | Second Discussion | Group |
| 13:15 | Tea and Coffee |  |
| 13:25 | Wrap up, final comments | Group |
| 13:45 | Expense forms | All |
| 14:00 | Close |  |

## Example Slide from Presentation
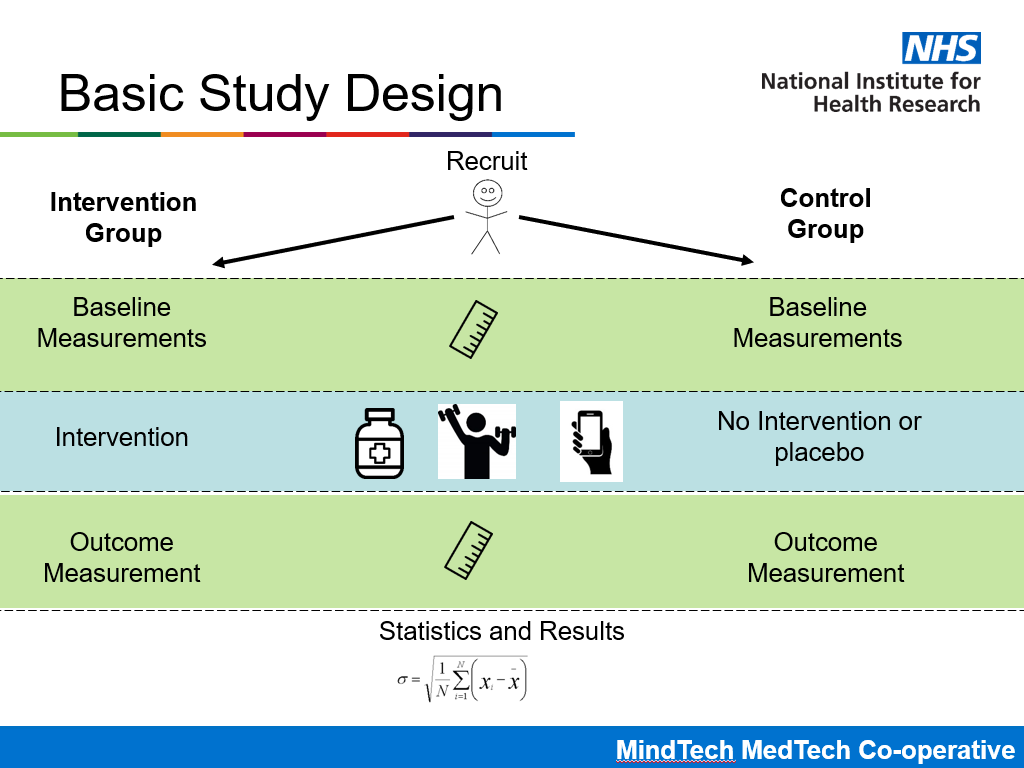


## Discussion Questions

- If you were designing a study to look for improvements in people’s cognition and/or health and quality of life, what sorts of things would you measure?
- How would you measure these things?
- Please feel free to make any suggestions you think of, we will be discussing these throughout the session. There are no wrong answers.

# Session 2 – 5/11/2019

## Timetable

| Time | Activity | People |
| --- | --- | --- |
| 09:45 | Team meet at IMH and walk over to room | Researchers and Facilitators |
| 10:00 | Group ensures room is ready. Information sheets, Expense forms, Consent Forms, Presentation | Researchers and Facilitators |
| 10:15 | Tea and coffees arrive. |  |
| 10:30 | Attendees arrive, given chance to read and sign consent forms. | Attendees |
| 10:40 | Introduction and thanks | Researchers |
| 10:45 | Welcome and Ground rules | Facilitators |
| 10:50 | Show and tell | Group Activity |
| 11:30 | Discussions around discussion point (1) | Activity |
| 12:00 | Lunch |  |
| 12:30 | What do we mean by intelligence? | Facilitator led discussion |
| 12:30 | Where do you stand? (2) | Group Activity |
| 12:50 | Discussions around discussion point (3) | Group Activity |
| 13:15 | Tea and coffee arrive |  |
| 13:20 | Closing remarks, comments – ideas for future sessions (4) | Group Activity |
| 13:40 | Expenses, paperwork | Facilitators to assist. |
| 13:50 | Thank you and close | Lead Researcher |

## Poster Advertisement


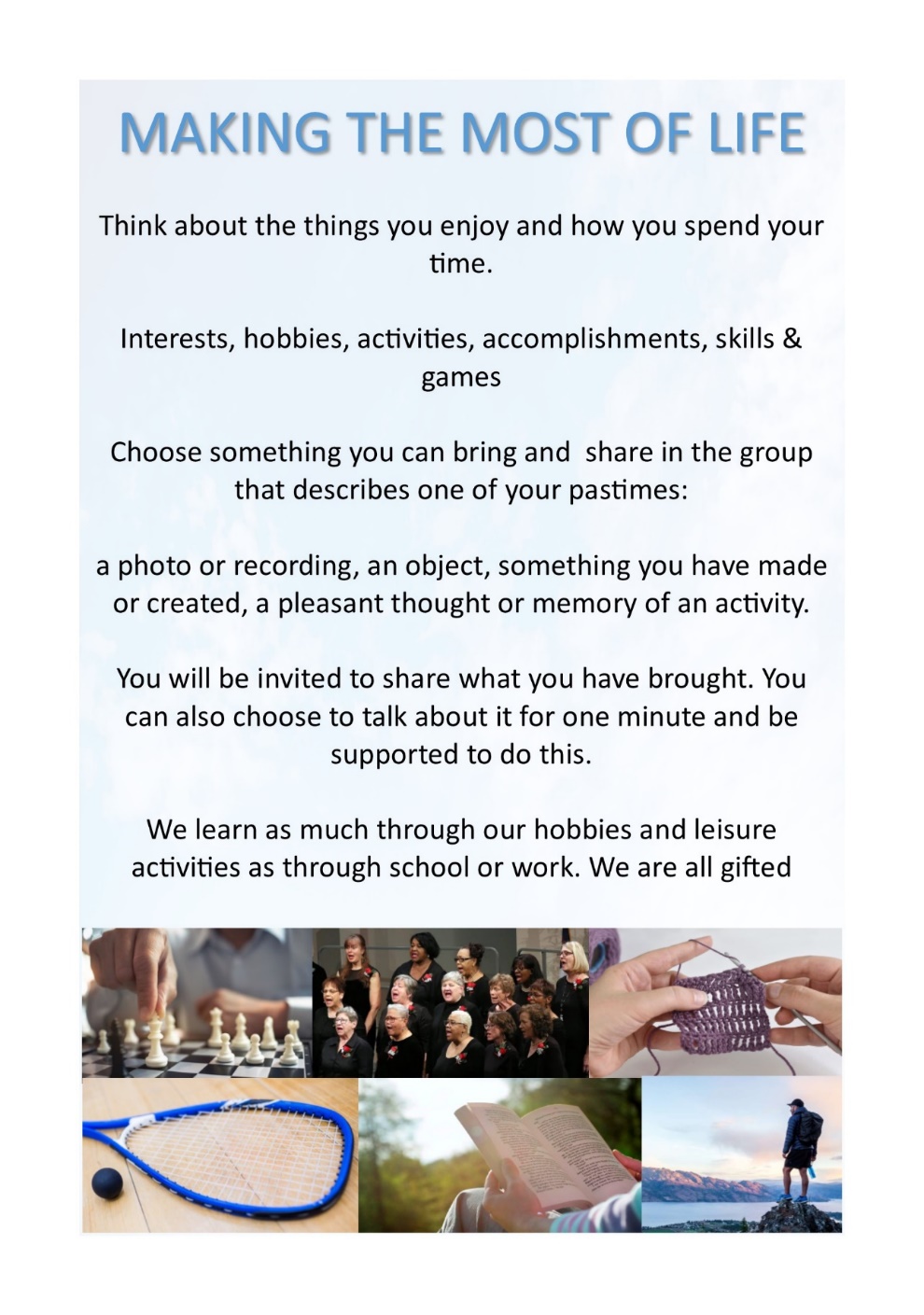


## Discussion point (1) Motivation

1. What motivates you to keep doing this?
2. How did you become interested in this?
3. What would stop you doing this?
4. Has anybody told you that you might not be able to do this activity in the future? Has anybody stopped you doing this now?
5. What factors which motivate you might be able to help motivate you in other areas?
6. How could people use some of the things we have discussed today to make Brain-Training games more motivating?

## Where do you stand? (2) Questions and Activity

1. The things I’m good at, I’m naturally good at vs the things I’m good at are because I have practiced them.
2. With support I can get better
3. I generally focus on my weaknesses vs I generally focus on my strengths.
4. I see my weaknesses as opportunities for growth vs I see my weaknesses as a threat
5. With enough practice, I could learn anything vs even with practice, there are certain things I would not be very good at.
6. With enough practice, I could make improvements, vs even with practice I would not be able to improve.

## Discussion Point (3) Learning

- How well can learn new skills as you grow older?
- What would help you to learn a new skill, habit or hobby?
- Are there any skills you’ve learnt later on in life? Computers, tablets, a second language, an instrument, a new hobby, knitting, sports.
- What were the important things that helped you learn this new skill?
- Was there anything that got in the way?
- How do you think lifelong learning can be encouraged? How have you been encouraged?
- How could technology help you to learn new skills?

## Closing Remarks, Comments, Ideas for future sessions

- Has anybody learnt anything today, or had their perspectives challenged?
- Have you learnt anything about yourself?
- Is there anything important that you think we’ve missed out?
- How could we make the next session better?

## Session 3 – 10/12/2019

## Timetable

| Time | Activity | People |
| --- | --- | --- |
| 10:30 | Room opens, participants sign consent forms | Everyone |
| 10:40 | Welcome and Introduction | Researchers and Facilitators |
| 10:45 | Discussing Diagnosis | Group Activity |
| 11:30 | Digital Screening Demonstration | Researcher 1 |
| 11:45 | Digital Diagnosis | All |
| 12:00 | Lunch | All |
| 12:30 | Conversation Facilitated by Ideation Cards | All |
| 13:00 | Further Discussions on Screening, Monitoring and Trust | All |
| 13:15 | Tea and Coffee Arrive | All |
| 13:30 | Concluding Remarks. A.O.B.  Discuss date for final group | All |
| 13:45 | Finish, expense forms | All |

## Discussing Diagnosis

1. Does anybody here feel comfortable in sharing their own experience of diagnosis?
2. Could you expand on whether you felt better or worse with after a diagnosis?
3. Before receiving your diagnosis, would you have liked to have known about your risk?
4. Are there any positive aspects of receiving a diagnosis?
5. How do you think support around a diagnosis could be improved?

## Digital Diagnosis

1. How would you feel about receiving information about your cognitive abilities via an app?
2. Would you be more or less likely to trust information you received from an app than a clinician?
3. How would you feel if an application made predications about your future cognition?
4. Is there anything which would make you trust an app more or less?
5. Would you be comfortable knowing that data about your cognition is being collected?

## Discussions for Future Development

1. If you were helping to design a digital screening tool, what would you think it was important to think about?
2. How could digital monitoring be improved?
3. Is there anything you think are particular risks for this type of technology?
4. I will go away from this meeting and tell Brain+ the sorts of things we have discussed today, is there anything you think is vitally important I communicate to them?

## Closing Remarks

1. Has anybody learnt anything today, or had their perspectives challenged?
2. Have you learnt anything about yourself?
3. Is there anything important that you think we’ve missed out?
4. How could we make the next session better?

# Session 4 -28/01/2019

## Timetable

| Time | Activity | People |
| --- | --- | --- |
| 9:45 | Facilitators check materials and plan | Researchers and Facilitators |
| 10:00 | Researcher Begins set-up of VR | External Researcher |
| 10:00 | Facilitators wait downstairs and see people to room | Facilitators |
| 10:00 | Participants start arriving |  |
| 10:15 | Tea and Coffee arrive |  |
| 10:30 | Session begins with welcome | Researchers and Facilitators |
| 10:35 | Icebreaker: What barriers did you overcome? | All |
| 10:50 | Personas and Vignettes | All |
| 12:00 | Lunch | All |
| 12:30 | Opportunities and Challenges | All |
| 13:15 | Tea and Coffees | All |
| 13:30 | Final Comments | All |
| 13:50 | Close |  |
| 14:00 | VR Demo | Researcher 4 and External Researcher |

## Example Personas


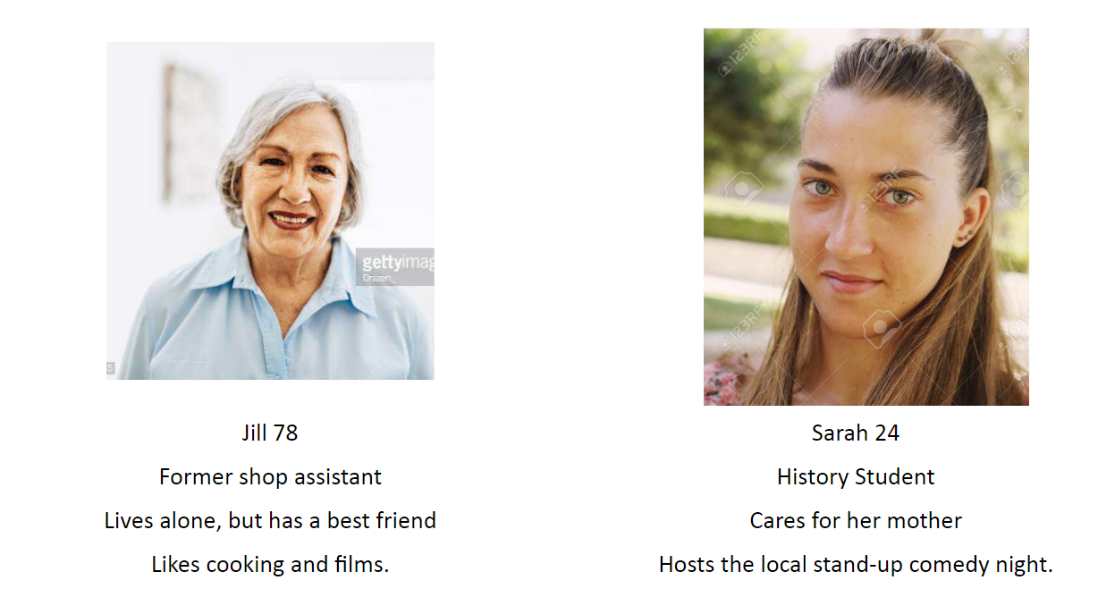


## Statements to discuss with personas

"I don't like to give my credit card information online."

“if I get stuck using my computer, I wouldn't know who to ask”

“I use technology to monitor my health.”

“I don’t know what information to trust”

“I enjoy video calling”

“I use navigation tools on my personal devices”

“I check reviews online before a decision”

# Certificate of Contribution


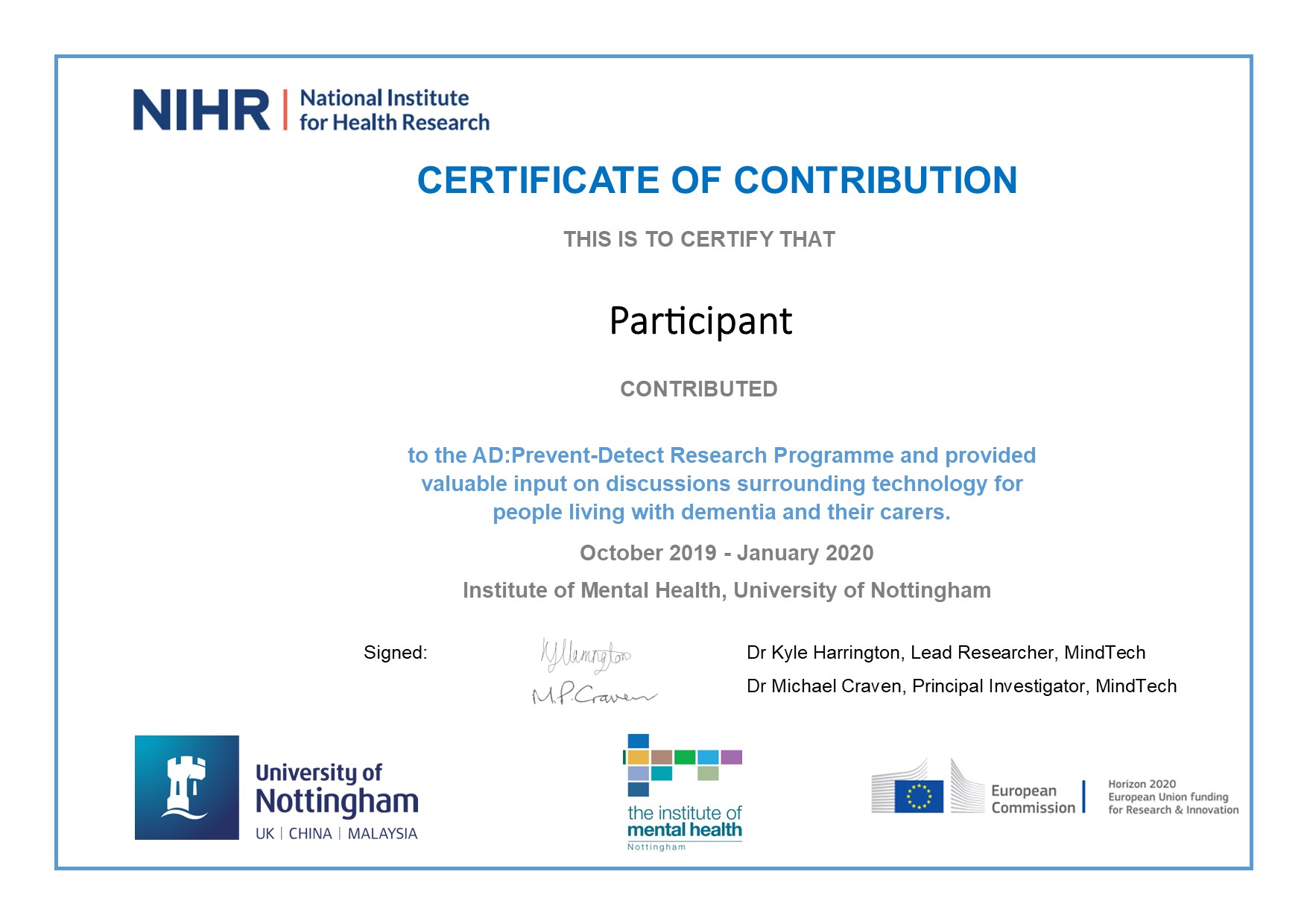


Focus Group Feedback Form

| Please tick where appropriate | Yes | No | Not Sure |
| --- | --- | --- | --- |
| I enjoyed the focus group |  |  |  |
| The focus group was well organised |  |  |  |
| I thought the activities were well-planned |  |  |  |
| I enjoyed discussing the topic |  |  |  |
| We were given enough time |  |  |  |
| The facilitators encouraged discussion |  |  |  |
| I got a chance to have my say |  |  |  |
| I felt listened to |  |  |  |
| I had the opportunity to take a break |  |  |  |
| The focus group was at a good pace |  |  |  |

Overall, the group was…

| Great | Good | Ok | Poor |
| --- | --- | --- | --- |
|  |  |  |  |

Was there something you think we should have discussed, but didn’t?

______________________________________________________________________________________________________________________________________________________

Any other comments?

______________________________________________________________________________________________________________________________________________________
