# Supplementary material for: Perceptions of Cognitive Training Games and Assessment Technologies for Dementia: Acceptability Study With Patient and Public Involvement Workshops
Source: JMIR Serious Games. 2022 Jun 20;10(2):e32489. doi: 10.2196/32489 (PMC9253969; doi:10.2196/32489)
Supplement: Multimedia Appendix 2 [file games_v10i2e32489_app2.docx]

Appendix II COM-B Themes

| Theme | Definition and Coding Guidance | Examples | No of Excerpts |
| --- | --- | --- | --- |
| **Capability** | Is a person capable of action? Do they have specific physical or cognitive issues which make this action more difficult? Do they struggle with the tasks of daily living? Is this person capable of using technology? Does this person have issues with their memory, navigation or planning? **Capability is related to what people are able to do.**  For the purposes of coding, codes being themed with capability must make an explicit reference, e.g., that a person has or does not have the capability to do something, not simply that something was done.  Related Concepts:  Ability, strengths, weaknesses. | *My main problem is I get too much information and too much going on and that confuses me completely and so much going on in newspapers*  *if I am going somewhere and I can’t turn in that road then I really panic – well I never used to panic but I do now.*  *I just tell myself I am getting old. But the words do come back but not at the time when you want it and you are struggling to get it out.*  *I think there also comes a time in your life when you can’t be as active as you used to be, and you possibly decide to do something which is less active, which is my case.* | 192 |
| **Opportunity** | Does the environment allow for such action? Environment could mean physical, or social. Are there any cultural norms which dictate or shape the environment? Does the person have a social life which influences this action, positively or negatively, making this action more of less likely? What is the technological environment like? What things do people have readily available access to? **Opportunity relates to what the environment encourages or discourages, and it relates to what is technologically possible.**  Related Concepts: Society, technology, the environment. | *there’s this lovely project where you join a choir, that’s a digital choir. So, you record your voice singing your bit, and then you start with five hundred in the choir, it was in like two thousand now. And this, whatever the process is, it brings all your voices together*  *I went to find out whether my GPs had it and we can, and so all the things I was doing on a computer, because I knew the computer things from years ago, I can do on the app now. So that’s prescriptions, make appointments, and you can look at results of tests as well.*  *I think were the intervention therapeutic it can be really helpful, but I think it's a thin end of the wedge where in certain conditions it might lead to some compulsory monitoring for example or tagging*  *You have to work twice as hard in order to get a level playing field. And I think that applies to race, it applies to gender, it applies to disability. You can just be; you have to prove yourself all the time.* | 391 |
| **Motivation** | Is this person inclined to perform such action? Does this person *want* to perform such an action? Do this person have either reflexive processes (plans, conscious wishes) or unreflexive processes (“involving emotions and impulses that arise from associative learning and/or innate dispositions”) that influence such behaviours? **Motivation relates to people’s dispositions towards this behaviour; their preferences and habits.**  Related Concepts: Likes, dislikes, hobbies, habits, plans. | *If it is stretching your brain, it must be a good thing.*  *I think positive feedback is good in any feel because I am doing this diet app at the moment and every day you get a little quiz and if I get 5 out of 5 I feel really good but if I get 2 wrong then you know I think oh I was not so good to day.*  *So, for me, positive feedback is really important.*  *So, whatever you are doing in Brain Training or whatever there needs to be a connection to the past because my past is very important to me, and your past is very important to you. But I need to be able to make connections with you, so that to be able to get there together*  *You don’t have to beat yourself up just because you can’t do it.* | 271 |
