# Supplementary material for: Perceptions of Cognitive Training Games and Assessment Technologies for Dementia: Acceptability Study With Patient and Public Involvement Workshops
Source: JMIR Serious Games. 2022 Jun 20;10(2):e32489. doi: 10.2196/32489 (PMC9253969; doi:10.2196/32489)
Supplement: Multimedia Appendix 3 [file games_v10i2e32489_app3.docx]

Appendix III Emergent Themes

| Theme | Definition and Coding Guidance | Examples | No of Excerpts |
| --- | --- | --- | --- |
| **Technology** | Comment references to technology, in particular – digital technology. | *For me, what is really useful to think about the internet is that we get access and curate. For example, music, films—so I’ve got my own repository now full of links and speaking books. You know, can get into it at the press of a button, it makes it so easy.*  *I contact my brothers in Canada. One of the things—I don’t see them all the time, so I use video call to talk to them.*  *I think computers are getting easier to understand and to work on. It's probably because we've started using them more and more* | 283 |
| **Friends, Family and Support Networks** | References to friends, family or wider support network. | *My husband knew before me but I carried on working and I managed to do my job until they had new IT and I couldn’t get to grips with it and that is when I knew it had started but he knew before me really.* | 177 |
| **Work and Hobbies** | References to work, occupation, or leisure activities (tennis, singing for the brain, etc) | *I see a great difference in this as my wife was able to sing and she loved the singing side, which is very well known with dementia*  *I have been hanging on to that because I have been learning Spanish for 10 years.*  *I found out that I had Alzheimer’s because I couldn’t do my job any more as a lawyer and I loved my job, and I loved all the people and suddenly I am at home now* | 244 |
| **Healthcare System** | Comments referring to medical appointments, treatments by primary, secondary or tertiary providers. | *If you contact your GP, so can get our prescriptions online. You know, I can make an appointment online, which is good.*  *If you contact your GP, so can get our prescriptions online. You know, I can make an appointment online, which is good.*  *They were training the future doctors, the doctors of the future: "This is what we need to hear from you." And that training, the best training, will come from people with dementia themselves, who will actually, you know, train the next generation of social workers, health workers, doctors, to say, "Please think, you know, if you were in my situation, what would you want to hear?"* | 154 |
| **Cognition** | Cognition shall be coded for if participants mention changes, impairments, or deficits in the cognitive abilities of somebody living with dementia. Also included is any mention of cognitive assessments, cognitive benefits of brain training activities or learnt cognitive strategies (e.g., mnemonics, calendars and reminders to compensate for memory problems). May also include confusion, difficulties with reasoning, memory and forgetting, information processing, planning, navigation, learning new skills or the ability to think clearly.  **Does not include dexterity or being able to perform complex motor functions quickly. Does not include general discussion of activities which require cognitive skills (driving, puzzle games), unless those cognitive skills, or cognitive assessments are explicitly the point of the excerpt. Does not include impact on the qualities of everyday living or ability to live independently unless cognition is mentioned or implied as the reason for the change.** | *I think it would be a situation where you could measure improvement if there are improvements because in really life there aren’t any it is the other way. So, improvements if there are such things would be measurable if there is one*  *I think from observation is that it is such a big change it is there, or it isn’t it is all or nothing. You can either can or can’t if you think of that as some sort of binary measures but what we hope from something like the games and also part of this app is about detection, you could have some kind of scales*  *We were walking round and round trying to find somewhere, he suddenly realised he had got the map upside down and that would never have happened, it was so out of character and there must of be lots of other little things, but I just thought there is something going on here that is not quite right.* | 189 |
